# Immunization against nucleus pulposus antigens to accelerate degenerative disc disease in a rabbit model

**DOI:** 10.3389/fvets.2024.1382652

**Published:** 2024-05-13

**Authors:** Andres F. Bonilla, Katie J. Sikes, Lindsey H. Burton, Lyndah Chow, Jade Kurihara, Kelly Santangelo, Steven W. Dow, Jeremiah T. Easley

**Affiliations:** ^1^Preclinical Surgical Research Laboratory, Department of Clinical Sciences, College of Veterinary Medicine and Biomedical Sciences, Colorado State University, Fort Collins, CO, United States; ^2^Immunotherapy Research Laboratory, Department of Clinical Sciences, College of Veterinary Medicine and Biomedical Sciences, Colorado State University, Fort Collins, CO, United States; ^3^Department of Microbiology, Immunology, and Pathology, College of Veterinary Medicine and Biomedical Sciences, Colorado State University, Fort Collins, CO, United States

**Keywords:** *in vivo*, model, spine, intervertebral disc, immune

## Abstract

Low back pain poses a significant societal burden, with progressive intervertebral disc degeneration (IDD) emerging as a pivotal contributor to chronic pain. Improved animal models of progressive IDD are needed to comprehensively investigate new diagnostic and therapeutic approaches to managing IDD. Recent studies underscore the immune system’s involvement in IDD, particularly with regards to the role of immune privileged tissues such as the nucleus pulposus (NP) becoming an immune targeting following initial disc injury. We therefore hypothesized that generating an active immune response against NP antigens with an NP vaccine could significantly accelerate and refine an IDD animal model triggered by mechanical puncture of the disc. To address this question, rabbits were immunized against NP antigens following disc puncture, and the impact on development of progressive IDD was assessed radiographically, functionally, and histologically compared between vaccinated and non-vaccinated animals over a 12-week period. Immune responses to NP antigens were assessed by ELISA and Western blot. We found that the vaccine elicited strong immune responses against NP antigens, including a dominant ~37 kD antigen. Histologic evaluation revealed increases IDD in animals that received the NP vaccine plus disc puncture, compared to disc puncture and vaccine only animals. Imaging evaluation evidenced a decrease in disc height index and higher scores of disc degeneration in animals after disc punctures and in those animals that received the NP vaccine in addition to disc puncture. These findings therefore indicate that it is possible to elicit immune responses against NP antigens in adult animals, and that these immune responses may contribute to accelerated development of IDD in a novel immune-induced and accelerated IDD model.

## 1 Introduction

Low back pain is a common multifactorial condition in the general population, causing a great economic impact due to loss of productivity and increased health care costs (1). Intervertebral disc degeneration (IDD) has been identified as one of the main causes of low back pain (2). Several animal models have been developed to understand the pathophysiology and evaluate new therapeutic strategies for low back pain caused by IDD (3, 4). However, there is scientific consensus around the need for improved animal models for elucidation of potential novel targets and approaches to treat IDD (5, 6).

The intervertebral disc (IVD) is the largest avascular structure in the human body. It is composed of an outer Annulus Fibrosus (AF), made up of concentric lamellae rich in collagen type I, and a central gelatinous nucleus pulposus (NP), rich in proteoglycans and collagen type II. With increasing age and degeneration, the disc tissue becomes disorganized (2). Specifically, irregularities and loss of hydration to the AF through aggrecan degradation occurs, which leads to progressive loss of space between vertebrae, and fissures of the AF fibers often leading to disc herniation and exposure of NP to the vascular system and subsequently to the immune system (7–9).

An immune component in the development of IDD has recently attracted the attention of several researchers (10–12). There are differing theories on the role that immunologically privileged features of certain tissues of intervertebral disc, most notably the NP antigens, play in the pathogenesis of the IDD (12–14). In healthy IVD, the NP is avascular and isolated from the immune system by the AF (15, 16). As IDD progresses, the vascularization process and fissures within the AF results in exposure of the NP antigens to cells and antibodies of the immune system. Degradation products of disc proteins can trigger an immune reaction experimentally, and there is evidence from clinical studies that IVD injury can induce the production of anti-NP antibodies (15, 17). Common techniques used to induce IDD in preclinical models involve disrupting the AF and exposing the NP antigens to the peripheral immune system. This exposure could trigger inflammatory responses within the IVD, thereby promoting further IVD injury (18). Several previous studies have reported finding anti-NP antibody and cellular responses in humans with IDD, as well as in animal models (17, 19–21). We hypothesized that administering a vaccine containing NP material would increase disc degeneration signs in an animal model compared to animals solely subjected to disc puncture without the vaccine. Our study is, to our knowledge, the first to combine inducing anti-NP immunity with mechanical IVD injury to accelerate IDD in an animal model. In this report, we present the results of our efforts to replicate and refine IDD in a rabbit model through vaccination against NP antigens combined with surgical IVD injury.

## 2 Materials and methods

### 2.1 Animals

This study was performed with approval from the Institutional Animal Care and Use Committee at Colorado State University (protocol#: 20-9762A). A total of 12 female New Zealand White rabbits (age range 12–16 months, weight range 4.3–5.2 kg) were acclimated at least 2 weeks prior study initiation. Animals were randomly assigned to NP-Vaccine group (NP-Vac), NP-Exposure group (NP-Exp) or NP-Vaccine + NP Exposure group (NP-Vac + NP-Exp) (*n* = 4 rabbits per group).

### 2.2 Vaccine preparation

Nucleus pulposus material was collected from two healthy sheep to prepare the NP antigens. Sheep were selected as a source of NP material because injected rabbits would likely have a higher chance of developing an immune reaction to NP proteins due to the species cross-reactivity. The NP tissue was then processed following the protocol proposed by Capossela (22). Briefly, the NP tissue samples were pulverized on dry ice in a stainless-steel mortar. Collagenase (Type 2, ThermoFisher Scientific) was added to cause digestion of the tissue, and lysates were homogenized using a Sonicator ultrasonic device. After 15 min of incubation at room temperature, lysates were centrifuged at 17,000 g for 15 min at 4°C to remove debris. Protein concentrations of supernatants were measured by BCA protein assay (Axonlab). To prepare the NP vaccine, NP proteins were mixed with a liposome-TLR agonist vaccine adjuvant, which has previously been reported to elicit high levels of both antibody and cellular immunity to a number of different protein and peptide antigens (23–25). The vaccine was developed under stringent conditions in a dedicated BSL2 laboratory. Each batch of the vaccine was uniquely derived from ovine nucleus pulposus extraction, ensuring consistency and uniformity across administrations. The rabbits were immunized with 250ug NP protein via the subcutaneous route, administered once every 2 weeks for a total of three immunizations, with the first dose being administered immediately before surgery.

### 2.3 Surgical intervention

All surgical procedures were conducted under aseptic conditions. Animals were pre-medicated with glycopyrrolate and buprenorphine. Once initially sedated using ketamine/dexmedetomidine, animals were placed on Isoflurane face mask at 4–5% until they reached a surgical plane of anesthesia, then maintained on 2–3% Isoflurane and 100% oxygen. Prior to surgery, each rabbit was placed in right lateral recumbency, and the posterolateral aspect (over the lumbar spine) was shaved and prepped using an alternating combination of 70% alcohol and chlorhexidine. For NP-Exp and NP-Vac + NP-Exp groups the lumbar spine was approached from the left side and the technique described by Luo et al. (26) was performed. Briefly, a minimally invasive transcutaneous needle puncture technique, guided by fluoroscopy, was employed using a 16G spinal needle to puncture the L2-3, L3-4, L4-5, and L5-6 intervertebral discs. Needle placement was confirmed through fluoroscopic images until it passed through the full diameter of the intervertebral disc without puncturing the contralateral annulus fibrosus.

### 2.4 Detection of anti-NP antibody responses by ELISA

Blood serum was collected prior to surgery and NP vaccine injection, and at 0, 2, 4, 8 and 12 weeks after these procedures to detect the presence of anti-NP antibody responses using an NP ELISA custom created for this study. Briefly, a 96-well Immulon plate (ThermoFisher) was coated with 100 μL of NP protein isolated from pooled sheep NP material collected at necropsy. The NP protein was diluted to a concentration of 20ug/mL in carbonate buffer. After incubation overnight at 4°C, wells were washed with PBS Tween using a plate washer, and non-specific binding sites were blocked for 2 h at room temperature with PBS + 10% BSA. After washing with PBS, 100 μL of rabbit serum (diluted 1:100 in 1%BSA/PBS) were added to the plates and incubated for 2 h at room temperature. After a second plate wash, 100 μL of 1:3000 dilution (in PBS + 1% BSA) of peroxidase conjugated donkey anti rabbit IgG (Jackson Immuno Research) was added and incubated for 1 h at room temperature. Following washing with PBS, TMB-ELISA Substrate Solution (ThermoFisher) was added and incubated for 10 min. Finally, 50 μL TMB stop solution was added and the optical density (OD) was measured at an absorbance at 450 nm. Optical density values were plotted, and pre-vaccination serum ODs were compared to post-vaccination ODs.

### 2.5 Western blot

The sheep NP proteins used to prepare the vaccine were also used in the Western blotting procedure. The NP proteins were prepared under reducing, denaturing conditions, and 20ug total protein was loaded into a 4–20% Mini-PROTEAN TGX gel (Bio-Rad) and transferred to PVDF membrane (Bio-Rad). After blocking the membrane with 5% BSA in Tris-buffered saline with 0.1% Tween (TBST) for 1 h, the membranes were then incubated with blood serum samples from 0 weeks and 12 weeks. Serums were diluted 1:100,000 using 5% BSA in TBST and incubated overnight at 4°C. The membranes were incubated 1 h at room temperature with Peroxidase AffiniPure^™^ Goat Anti-Rabbit IgG (H + L) (Jackson Immunoresearch, United States), followed by washing, the blots were developed using Clarity Western ECL Substrate (Bio-Rad) and imaged on ChemiDoc XRS+ with Image Lab Software (Bio-Rad). Bands of gel that showed robust antibody response were isolated and submitted for proteomic evaluation using Mass spectrometry analysis (Orbitrap Eclipse, Thermo Scientific). Raw data was evaluated using Proteome Discoverer 3.0 (Thermo Scientific) and interrogated against the FASTA reference proteome of *Oryctolagus cuniculus* (rabbit, taxon ID 9986) from Uniprot. Additionally, the cRAP proteome was included (The common Repository of Adventitious Proteins -cRAP- contains commonly found contaminant proteins in proteomics experiments).

### 2.6 *In vivo* imaging

**Figure 1:**
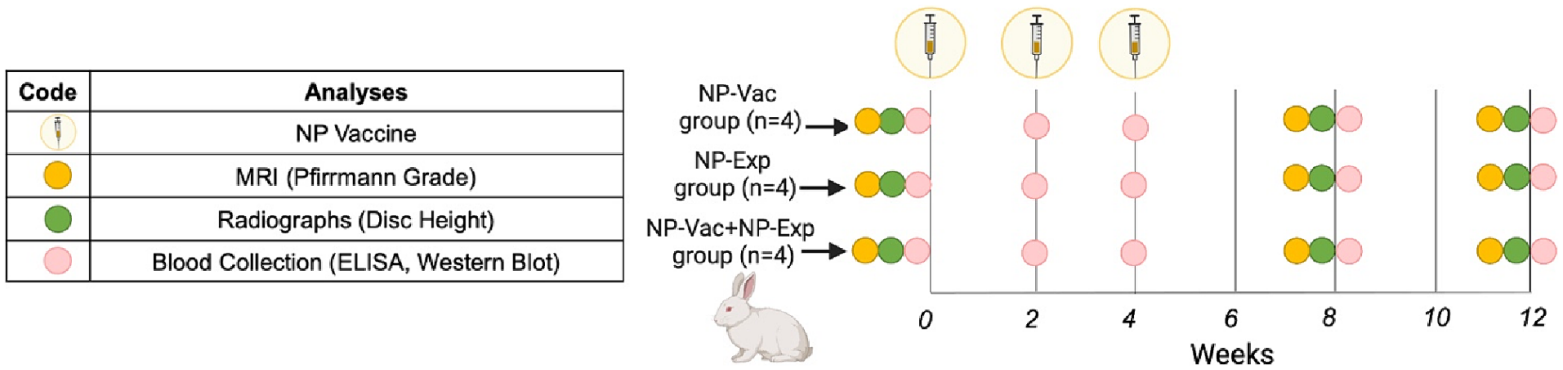
Schematic representation of the study design. Rabbits were allocated into three groups, NP-Vaccine group (NP-Vac), NP-Exposure group (NP-Exp) or NP-Vaccine + NP Exposure group (NP-Vac + NP-Exp). NP vaccine (syringe) was administered at 0, 2, and 4 weeks for NP-Vac and NP-Vac + NP-Exp groups. Imaging, comprising radiographs and MRI, was conducted at 0, 8, and 12 weeks (Yellow and green circles, respectively). Subsequent blood collection for ELISA and Western blot evaluation took place at 0, 2, 4, 8, and 12 weeks (Pink circles).

Radiographs and MR (Magnetic Resonance) imaging of the lumbar spines were performed prior to injury, 8 weeks post-injury, and immediately pre-mortem at 12 weeks (Figure 1). Animals were pre-medicated and anesthetized by initial induction of 4% isoflurane, with maintenance at 1–3%, and placed in the prone position. Lateral and dorsoventral digital radiographic views of the lumbar spines were acquired for each animal and use to evaluated significant bone abnormalities. The MR imaging was performed using a 3T MR scanner (Siemens 3T MAGNETOM Skyra MR Scanner) to obtain 2-dimensional T1 and T2-weighted sequences in sagittal orientation, and axial views with a T2-weighted sequence. The following basic protocol parameters were used for image acquisition: RT 3010 ms, ET 97 ms, 1.5 mm slices, acquisition matrix 384 × 288, Flip Angle of 260 degrees, and bandwidth of 480 Hz. Evaluation of MR images was completed by two blinded observers (AB, JE) and used to determine disc height index (DHI) (27, 28), and Pfirrmann grade (29). MRI was chosen to assess DHI due to its superior resolution, which enables precise evaluation of disc height changes within the same slice or plane, enhancing the accuracy of measurements and analysis.

### 2.7 Euthanasia and sample harvest

The rabbits were humanely euthanized 12 weeks after surgery and/or first vaccination by intravenous overdose of pentobarbitone sodium (88 mg/kg), in accordance with the American Veterinary Medical Association (AVMA) guidelines. The lumbar spines were removed to complete the *ex vivo* evaluation. Additional evaluation methods, including Micro-CT, biomechanical testing, biochemical assays (L3-4, L5-6), and histomorphometry, along with corresponding results, are detailed in the Supplementary material.

### 2.8 Histopathological analysis

Two functional spinal units (L_2-3_, L_4-5_) were bisected in the sagittal plane and processed for decalcified histological analysis. Following fixation, specimens were decalcified using EDTA 10%. Then, specimens were processed using standard techniques (Tissue-Tek VIP, Sakura, Torrance, CA) and embedded in paraffin. Two slides were produced from each sample. One was stained with Hematoxylin Eosin and the other with Alcian blue for evaluation of glycosaminoglycans (GAG). Histology sections stained for analysis underwent meticulous evaluation by two blinded observers, one being a certified veterinary pathologist. Utilizing a specific scoring system tailored for IDD in rabbit models (30), various parameters including nucleus pulposus (NP) morphology (shape and area), NP matrix integrity, NP cellularity, distinction of annulus fibrosus (AF) and NP border, AF morphology, and endplate (EP) thickening were assessed.

### 2.9 Statistical analysis

Following data processing, statistical analyses were performed on all outcome parameters. Standard two-way ANOVA test, followed by Tukey’s multiple comparison test, was performed to determine statistically significant differences (*p* ≤ 0.05) within and across treatment groups (GraphPad Prism 8.3.0, San Diego, CA). Data for ELISA, histological grading, Pfirrmann grade, and disc height are presented as mean ± SD.

## 3 Results

### 3.1 Assessment of anti-NP immunity using ELISA screening

**Figure 2:**
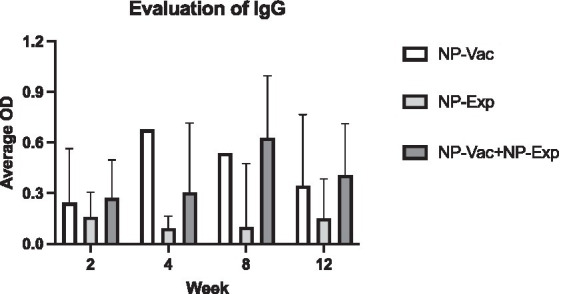
Evaluation of IgG in animals after induction of IVD degeneration. Animals from NP-Vac + NP-Exp group showed higher levels of IgG at 2-weeks, 4-weeks, 8-weeks, and 12-weeks compared with animals from NP-Vac and NP-Exp groups. An increase trend in IgG in the NP-Exp in time is also evident after exposure of the NP for NP-Exp group. Data are presented as mean with SD for each group with values normalized to 0 and excluding negative values; OD, Optical density.

We assessed the impact of NP vaccination on induction of anti-NP antibody responses to determine the effectiveness of the NP vaccine. Serum samples from animals in all three study groups were evaluated for the presence of anti-NP antibodies using an NP ELISA (see Methods). In both groups receiving the NP vaccine, strong IgG antibody responses to NP antigens developed and were detectable after the first immunization, increasing further by weeks 8 and 12 (Figure 2). Conversely, anti-NP antibody responses were not detected in the control or disc puncture only groups of animals. Notably, antibody responses were detected at numerically higher in the NP vaccine plus disc puncture group of animals, though the differences in antibody responses between the vaccine only group and the vaccine plus disc puncture groups did not reach the level of statistical significance. These findings indicate, therefore, that the NP vaccine effectively elicited rabbit humoral immune responses against NP.

### 3.2 Western blot

**Figure 3:**
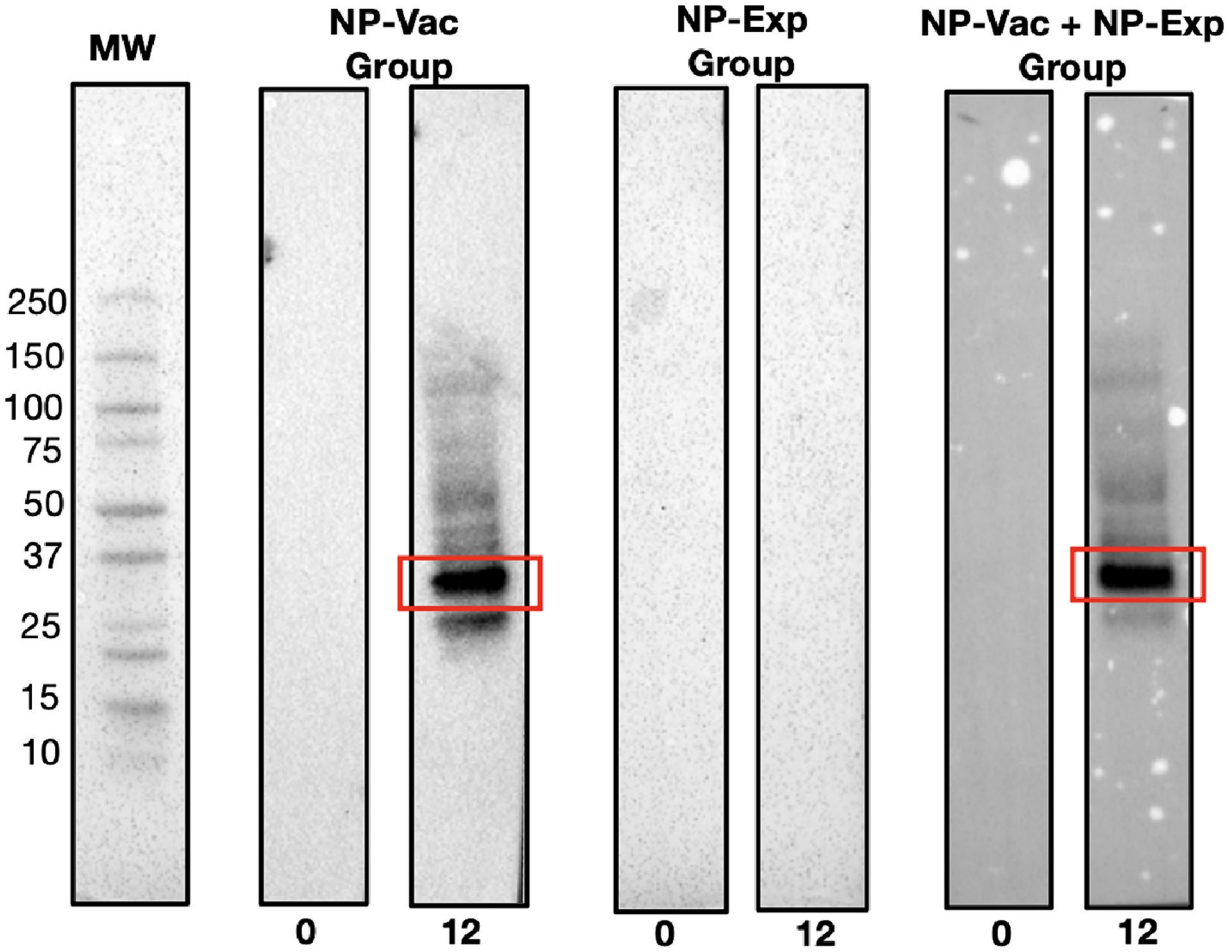
Representative Western blot results demonstrating distinct immunoreactivity patterns among experimental groups. Clear bands at the 12-week time point in lanes corresponding to the NP-Vac group and the NP-Vac + NP-Exp group indicate a robust antibody response post-vaccination (Red squares). In contrast, no observable bands are seen in the lane representing NP-Exp group, emphasizing the lack of immunoreactivity in this group. Results of all groups are presented in the Supplementary material; MW, Molecular weight.

Next, we used Western blotting to identify dominant NP antigens being recognized by the anti-NP antibody responses. Intriguingly, in both groups of NP vaccinated animals, there was strong antibody recognition (Figure 3). Proteomic analysis of the bands exhibiting strong antibody response revealed the presence of 193 proteins and 958 peptides. Table 1 presents the most abundant proteins identified, which correlate with the molecular weights observed in the Western blot results. Therefore, evidence from this study suggests that few highly immunogenic NP proteins may be targets for immune recognition in cases of naturally occurring IDD. Identifying the nature of these antigens is important for better understanding IDD pathogenesis and designing potential therapeutic interventions in patients with IDD.

**Table 1:** Most abundant proteins with similar molecular weights to those observed in Western blot results.

Accession	Description	Molecular weight (kDa)
G1SHY5	Hyaluronan and proteoglycan link protein 1	40.2
A0A5F9DKI8	Actin, cytoplasmic 2	40
G1T5H0	HtrA serine peptidase 1	36.4

### 3.3 Histopathological analysis of IVD tissues and impact of NP vaccination

**Figure 4:**
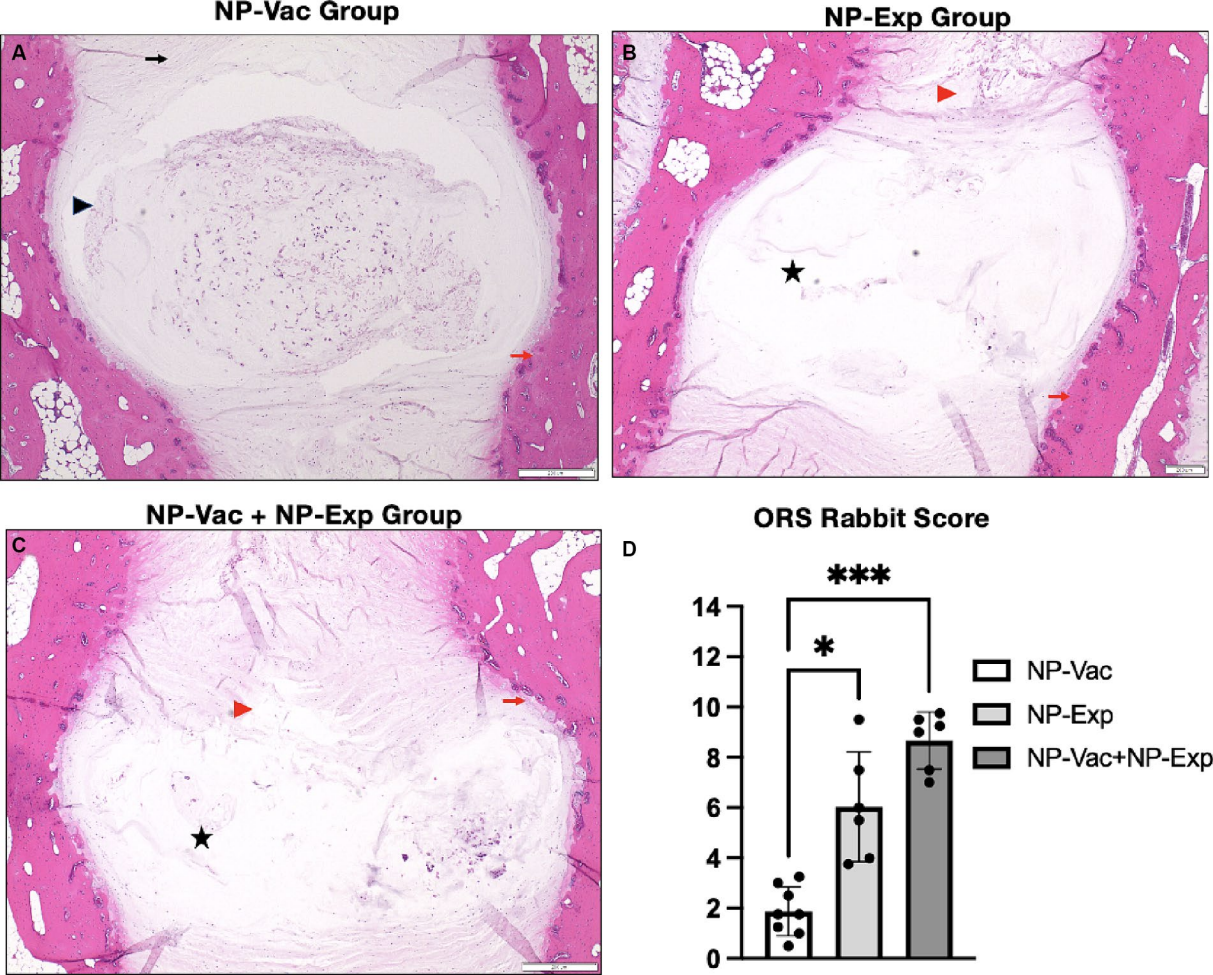
Representative histological images of the IVD and histopathological scoring from NP-Vac, NP-Exp, and NP-Vac + NP-Exp groups. **(A)** Animals from NP-Vac group showed clear NP shape (Black arrowhead) and good integrity of the AF area (Black arrow), and sclerosis of the endplates (Red arrows). Conversely, NP-Exp **(B)** and NP-Vac + NP-Exp **(C)** groups showed irregular NP shape area, and less cellularity (Black star), and loss of distinction between NP and AF (Red arrowhead). Subjective histological assessment showed increased IVD degeneration in NP-Vac + NP-Exp animals compared with NP-Vac group or NP-Exp animals. **(D)** Objective blinded histological scoring showed a significantly higher degree of IDD for NP-Vac + NP-Exp group animals compared with NP-Vac group (*p* < 0.05); NP, Nucleus pulposus; AF, Annulus Fibrosus; H&E, scale bar: 200 μm.

All three groups of animals in the study showed varying signs of IDD development, involving the AF, NP, and endplates, compared to healthy control reference values (30). Varying degrees of loss of distinction of structural components of the disc were evident in all three groups over the 12-week study period. Animals from NP-Vac group showed clear NP shape and evident integrity of the AF area but sclerosis of the endplates, compared with NP-Exp and NP-Vac + NP-Exp groups. In contrast, these latter two groups exhibited irregular NP shape and area, loss of distinction between NP and AF, and sclerosis of the endplates. Histological assessment showed increased severity of IDD in the NP-Vac + NP-Exp group compared with both the NP-Vac or NP-Exp groups, as well as with healthy control references (30). Histological scoring for rabbit IVDs showed a significantly higher degree of IDD, specifically for NP-Vac + NP-Exp animals compared with NP-Vac group (*p* = 0.339) (Figure 4).

### 3.4 Imaging of IVD injury sites following surgery and NP vaccination

**Figure 5:**
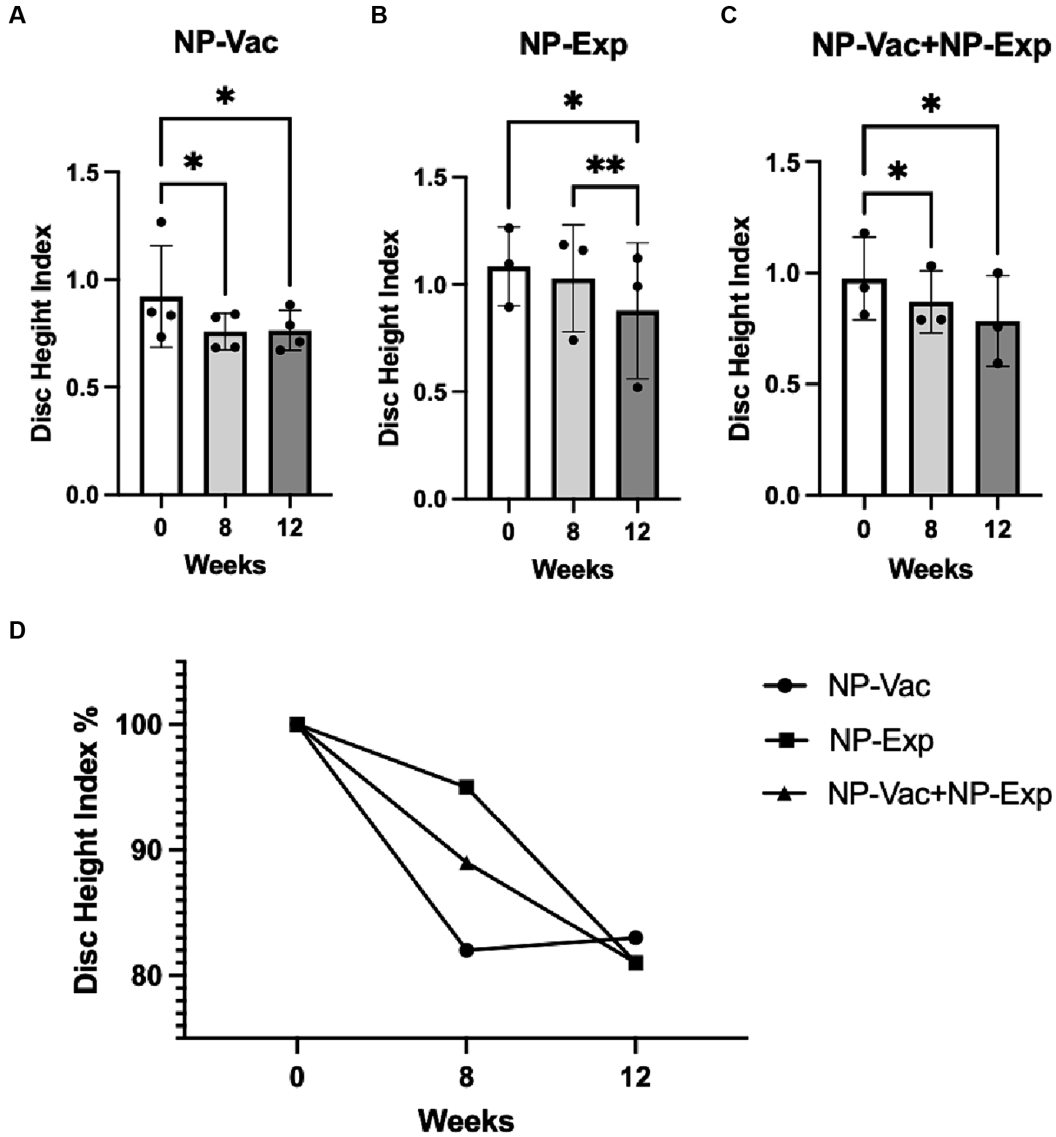
Changes in disc height index (DHI) for all experimental groups. **(A)** NP-Vac group showed significant changes between baseline (0 weeks), 8 and 12 weeks. **(B)** NP-Exp group showed significant differences in the DHI between 0 and 12 weeks, and between 8 and 12 weeks. **(C)** NP-Vac + NP-Exp group showed significant differences in the DHI between 0 and 8 weeks, and between 0 and 12 weeks. **(D)** Noticeable reduction in DHI is evident across all experimental groups, indicating a consistent trend of disc height decrease over time; * = *p* < 0.05, ** = *p* < 0.01; DHI, Disc height index.

Evaluation of the DHI showed a significant decrease for all groups after surgical intervention (disc puncture). The NP-Vac group showed a decrease in DHI at 8 weeks (*p* = 0.0255) and 12 weeks (*p* = 0.0119) compared to baseline. Animals from the NP-Exp group exhibited a decrease in DHI between 0 and 12 weeks (*p* = 0.0254) and between 8 and 12 weeks (*p* = 0.0076). Animals from NP-Vac + NP-Exp showed a decrease in DHI at 8 weeks (*p* = 0.0420) and 12 weeks (*p* = 0.0398) compared to baseline. The comparison of DHI between groups showed a decrease in DHI across all groups, although no significant differences were observed between them (Figure 5). Four animals (two from NP-Vac group, one from NP-Exp group, and one from NP-Vac + NP-Exp group) showed signs of IDD in the MR images at baseline (Average of Pfirrmann grade of the 4 lumbar disc levels = 1.5, 2.625, 1.367 and 2.875 respectively).

**Figure 6:**
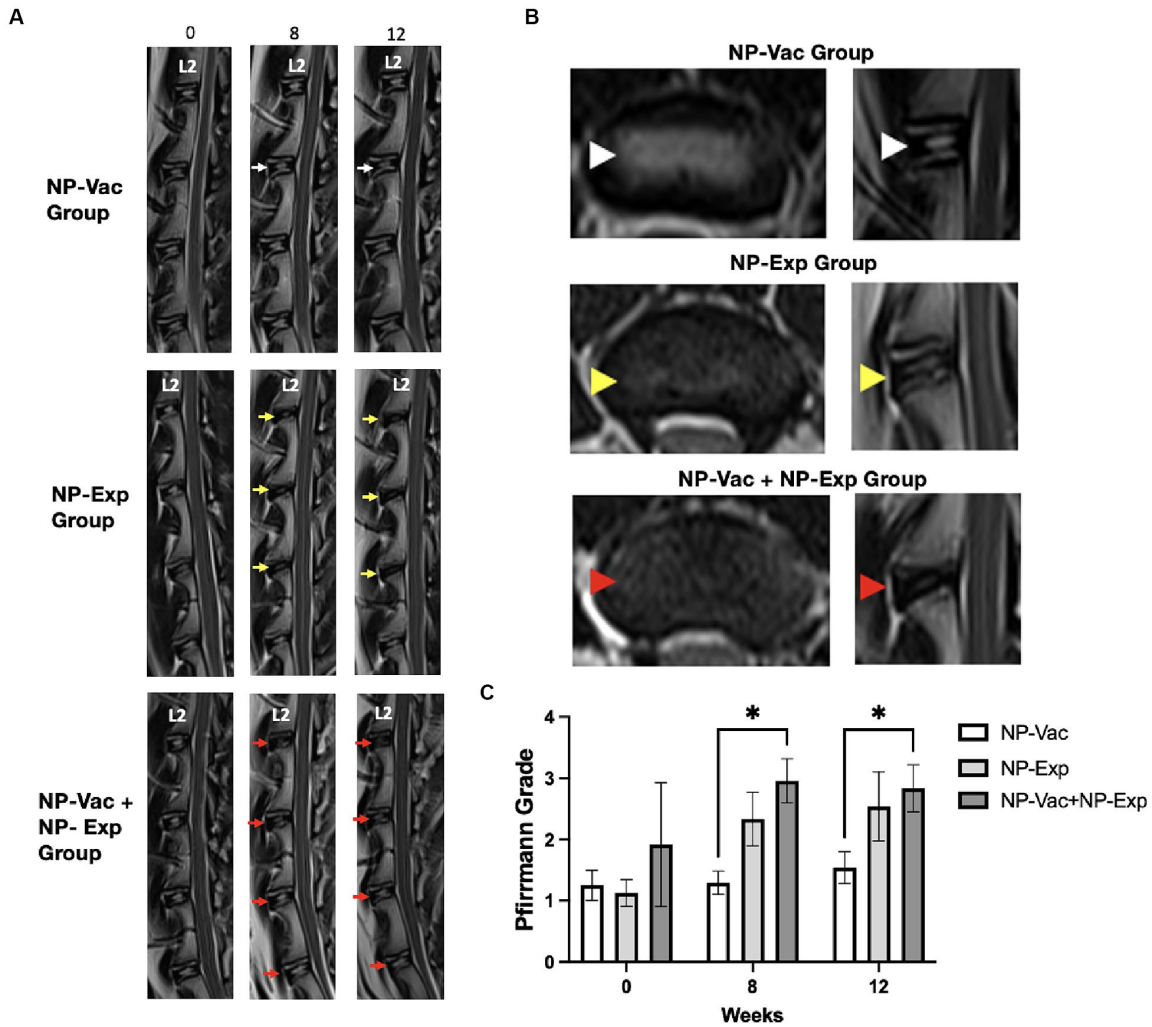
Magnetic resonance (MR) imaging evaluation showed higher degeneration grade for animals from NP-Exp group and NP-Vac + NP-Exp compared to NP-Vac. **(A)** Representative T2-weighted MR images from each group. NP-Exp and NP-Vac + NP-Exp groups showed more drastic decreases in signal intensity and increases in Pfirrmann grade defect types (Yellow and red arrows respectively) compared to NP-Vac group (White arrows) at 8 or 12-weeks. **(B)** Comparison of Axial and Sagittal T2-weighted MR views from the same disc in representative animals from each group at 12 week time point. Normal signal intensity of the NP is evident in NP-Vac group (white arrow heads) but decreased in NP-Exp group (Yellow arrow heads) and even more in NP-Vac + NP-Exp group (Red arrow heads). **(C)** Evaluation of the Pfirrmann grade showed significant differences with a higher degree of degeneration in animals from NP-Vac + NP-Exp group compared with NP-Vac group, after 8 and 12 weeks of intervention; * = *p* < 0.05. Data are presented as mean and SD.

Magnetic resonance imaging was also used to evaluate the impact of surgical disc disruption alone or in combination with an NP vaccine in the rabbit model. We found that MR and radiograph imaging did not detect evidence of new bone formation (osteophytes) or abnormalities after NP vaccination or vaccination plus disc puncture in any of the injured disc spaces from any of the three groups of animals. MR images from NP-Vac group of animals showed consistent homogeneous high-signal intensity within the central region of the IVD over the 12-week study period, indicative of healthy IVD. In contrast, MR images from the NP-Exp and NP-Vac + NP-Exp groups showed a significant decrease in signal intensity within the central region of the IVD, indicative of moderate IDD. No significant differences were noted in Pfirrmann grades at either 8 or 12 weeks between NP-Vac and NP-Exp groups. However, Pfirrmann grades were significantly higher in the NP-Vac + NP-Exp group compared to the NP-Vac group at 8 weeks (*p* = 0.0114) and 12 weeks (*p* = 0.0241) (Figure 6). These findings are consistent with the acceleration of disc injury in the animals that received the NP vaccine plus disc puncture surgery.

## 4 Discussion

This study, to our knowledge, is the first to provide evidence that immunization against NP antigens can refine and/or accelerate IDD in an animal model. Key findings from this study were induction of strong immune responses against a dominant NP antigen by NP vaccination and the progression of disc degeneration parameters in animals that received the NP vaccine plus surgical disc puncture. Our findings also indicate that this new animal model of accelerated IVD damage by immune processes could be an important new tool to elucidate the role of immune responses in human IDD and to develop new medical or surgical interventions to ameliorate anti-NP immunity (31). These new findings suggest that indeed anti-NP immune responses may be an important part of the progressive nature of IDD in animal models and that immune interventions designed to blunt these immune responses may be one strategy to slow or reverse IVD progressive damage.

The potential role of the immune system in the development and progression of IDD was introduced several decades ago (16, 32). *In vitro* studies support the notion that both degenerated and normal NP proteins can elicit detectable immune responses (17). Additionally, clinical findings by Kim et al. (32) demonstrated increased anti-NP antibodies in patients with spontaneous disc herniation. Satoh et al. (21) also identified antigen–antibody complexes in herniated NP tissue compared to non-herniated NP tissue. Capossela et al. (22) detected antibody responses against specific proteins in the NP in patients with IVD, confirming an immune reaction against the immune-privileged NP antigens.

In this study, we offer an insight into the proteins recognized by the NP vaccine. HtrA serine peptidase and Hyaluronan and proteoglycan link protein 1 (HAPLN1) proteins may serve as potential targets as they were both abundantly detected in the Western blots reacting to NP vaccine serums. HAPLN1 has been implicated in the degradation of the extracellular matrix and the process of intervertebral disc degeneration in both humans and animals (33–35). Similarly, HtrA serine peptidase is recognized for its involvement in osteoarthritic pathology and intervertebral disc degradation. Notably, elevated HTRA1 mRNA expression in degenerated disc tissue has been associated with the promotion of IVD degeneration through the proteolytic cleavage of fibronectin and subsequent activation of resident disc cells (36).

We found that animals that received the NP vaccine and then underwent disc puncture developed stronger antibody responses than animals that only received the NP vaccine. This finding would therefore be consistent with exposure of NP proteins to the immune system by virtue of physical barrier disruption (AF/disc puncture), which would serve to accelerate anti-NP immunity induced by the NP vaccine. While the literature primarily focuses on anti-NP immunity in disc herniations, our rationale for utilizing healthy NP tissue stems from its ability to serve as a reliable antigen source while minimizing potential complications associated with diseased tissue. Additionally, previous studies have demonstrated *in vitro* immune reactions to healthy NP cells (20), suggesting their suitability for stimulating an immune response in the context of vaccine development. It is also important to note here that the NP vaccine used in this study was derived from sheep NP material and was therefore immunologically foreign to rabbits in this study, which likely resulted in enhanced immune recognition, compared to immunization against rabbit NP proteins. It would be important to determine in subsequent studies whether immunization with autologous NP proteins derived from rabbit NP material could also induce strong anti-NP antibody responses in rabbits. Our intriguing early findings nonetheless suggest that the exposure of the NP to the immune system by needle puncture, coupled with the administration of an NP vaccine, can generate an enhanced immune reaction, as reflected by higher antibody titers in animals receiving disc puncture and the NP vaccine.

With respect to morphological markers of IDD, we noted a significant decrease in DHI across all three treatment groups, as reflected by sequential imaging. Intriguingly, even though the IVDs in the NP-Vac group were not punctured, baseline MR images revealed that these animals already displayed spontaneous signs of IDD. The observed spontaneous IDD changes at baseline align with previous reports indicating age-dependent alterations in intervertebral discs among rabbits (37, 38). This apparent naturally occurring IDD, combined with the immune effects of the NP-Vac, might account for an increase in IDD-related inflammation, leading to a reduction in the DHI for this group. While the NP-Exp (disc puncture) group showed consistent and expected MR changes and Pfirrmann grades to previous studies in rabbits (39–41), NP-Vac + NP-Exp resulted in more severe decreases in signal intensity within the disc. The increase in Pfirrmann grades of the animals from NP-Vac + NP-Exp group at 8 weeks, without significant changes at 12 weeks, suggest that a shortened study of only 8 weeks could be used for the establishment of the proposed accelerated and sustained model of IDD. Developing a more effective model to induce IDD would hasten the currently prolonged periods of time required for IDD progression, thereby reducing model time and costs. Furthermore, identifying and suppressing this immune response could also be used to decrease the development and progression of IDD in patients affected by the condition (31, 42, 43).

With the progression of IDD, the structure of the IVD becomes disorganized. Histologically, there is loss of distinction between the NP and AF, a reduction in cell density, and NP shape, and a progressive disorganization of AF. Our results show similar histological signs of IDD progression to previously reported IDD in rabbits with different methods to induce IDD (44). However, higher values of degeneration were observed in the NP-Vac + NP-Exp group using the standardized histopathology scoring system for IDD in rabbit models. This supports our hypothesis that the exposure of NP, in addition to the immune effects of receiving the NP vaccine, induced more severe changes compared to IDD induced through needle puncture alone.

The small sample size is a major limitation of this study, as is the lack of a control group of animals that received no interventions. Nevertheless, despite the limited number of animals in this study, we were able to identify evidence of a treatment effect from NP vaccination combined with surgical disc puncture. Therefore, we propose that there was sufficient evidence of immune activation of the IDD process in this new animal model to warrant further investigation, including studies with larger groups and additional controls, and with an optimized NP vaccine protocol.

Finally, the NP vaccine antibody results combined with significant changes in the Pfirrmann grade and histological evaluation, provide compelling evidence demonstrating the influence of the immune response on the development of IDD in a new *in vivo* model for IDD. In general, these results support our hypothesis that rabbits vaccinated with NP proteins developed a heightened immune response and increased IDD compared to rabbits undergoing IDD via a traditional needle puncture approach. However, further studies are imperative to gain a more comprehensive understanding of the role played by immune responses, particularly the relative importance of humoral responses (measured in the current study) and cellular responses (e.g., T cell responses) in the overall development of accelerated and sustained disc degeneration.

## Data availability statement

The raw data supporting the conclusions of this article will be made available by the authors, without undue reservation.

## Ethics statement

The animal study was approved by Institutional Animal Care and Use Committee at Colorado State University (protocol#: 20-9762A). The study was conducted in accordance with the local legislation and institutional requirements.

## Author contributions

AB: Conceptualization, Investigation, Methodology, Writing – original draft, Writing – review & editing. KaS: Investigation, Methodology, Writing – review & editing. LB: Investigation, Methodology, Writing – review & editing. LC: Investigation, Methodology, Writing – review & editing. JK: Investigation, Methodology, Writing – review & editing. KeS: Methodology, Writing – review & editing. SD: Conceptualization, Writing – original draft. JE: Conceptualization, Investigation, Methodology, Writing – original draft.
